# “We Learn How to Become Good Men”: Working with Male Allies to Prevent Violence against Women and Girls in Urban Informal Settlements in Mumbai, India

**DOI:** 10.1177/1097184X18806544

**Published:** 2018-10-18

**Authors:** Proshant Chakraborty, David Osrin, Nayreen Daruwalla

**Affiliations:** 1 Society for Nutrition, Education, and Health Action (SNEHA), Dharavi, Mumbai, India; 2 Institute for Global Health, London, United Kingdom

**Keywords:** engaging men, hegemonic masculinity, male allies, urban informal settlements, violence against women and girls, violence prevention

## Abstract

Engaging men has now become part of established global efforts to prevent violence against women and girls (VAWG), with most interventions focusing on making men’s behaviors and attitudes more gender equitable. While scholarship on male allies has demonstrated the nature of their transformations and motivations, less attention has been paid to their negotiations of masculinity, privilege, the intersection between subjecthood and social contexts, and how these inform their engagements with women activists’ anti-violence work in their communities. We explore questions of men’s engagement in this article, which is based on a pilot ethnographic study with male allies in a VAWG prevention program in the informal settlements of Dharavi in Mumbai, India. We found that while men are able to acquire “knowledge” and “awareness” through the intervention, it produces an individuating effect wherein the structural nature of VAWG is obscured due to an emphasis on men’s individual traits. This further informs participants’ understanding of masculinity, which is marked by ambivalence as men negotiate multiple hegemonic masculinities and socioeconomic anxieties. One reason for this is that interventions with men are unable to destabilize public–private boundaries in informal settlements, which continue to treat VAWG as “private matters.” We discuss the implications for local and global responses to engender accountability among male allies.

Violence against women and girls (VAWG) and other forms of gender-based violence (GBV) have been high on the global feminist and developmental agenda for several decades and are important areas for activist intervention. Responses include crisis, counseling, and legal support for survivors; community-based programs for advocacy and intervention by frontline workers; and education and consciousness-raising activities with survivors and communities (Coomaraswamy and Perera-Rajasingham 2008; Dobash and Dobash 1992; Kumaran 2014; Wies and Haldane 2015). While a mainstay of such interventions has been to work closely with women—survivors of violence and others—to enable them to prevent VAWG in their homes and communities, the fact remains that it is by and large men who commit such violence, and much of it takes place in intimate and familial relationships (Barker, Ricardo, and Nascimento 2007; Kaufman 1987; Fulu et al. 2013; Peacock and Barker 2014; World Health Organization [WHO] 2013).

Feminists, gender studies scholars, and activists have long argued that sociocultural and political constructions of hegemonic masculinity and patriarchal social norms underpin violence against women and other gendered bodies (such as nonbinary or transgender individuals) at interpersonal and structural levels (Brod and Kaufman 1994; Connell 2005; Connell and Messerschmidt 2005; Kaufman 1987; Flood 2011). Masculinity and masculine behavior—particularly violence—are understood within cultural and sociopolitical systems of domination rather than as a product of biological difference (Kaufman 1987), and efforts to prevent violence must acknowledge this to produce credible and sustainable change (Flood 2006). Masculine behavior and patriarchal social norms are manifest as risks of bodily injury, sexually transmitted infection, and VAWG, including sexual and domestic abuse and intimate partner violence (IPV; Brod and Kaufman 1994; Kaufman 1987; Fulu et al. 2013). Worldwide, almost one-third (30 percent) of women who have been in a relationship report that they have experienced some form of physical or sexual violence by an intimate partner in their lifetime (WHO 2013). Data from the 2010 International Men and Gender Equality Survey show that men’s reported IPV rates are highest in Rwanda (39 percent) and India (37 percent; Barker et al. 2011, 43–44). In India, according to the fourth and latest round of the National Family Health Survey, 29 percent women have ever experienced spousal abuse (Indian Institute of Population Sciences & Ministry of Health and Family Welfare [IIPS and MoFHW] 2017).

There is, therefore, an increasing awareness within the field of VAWG prevention that, in order to bring about sustainable change and achieve gender equality and justice, it is also important to work with men across communities, including those who are currently nonviolent and nonabusive (Berkowitz 2004; Barker, Ricardo, and Nascimento 2007; E. Casey and Smith 2010; Flood 2006; Peacock and Barker 2014). The field of men’s and masculinity studies has grown considerably since the 1980s (Brod and Kaufman 1994; Chopra 2007; Connell 2005; Kaufman 1987), but global research on engaging men in violence prevention has been sparse until the last decade, most research coming from feminist and women’s groups and nongovernment organizations (NGOs). Recent studies on men’s involvement in anti-violence work and allyship bridge this gap and suggest directions for further research (Carlson et al. 2015; E. Casey and Smith 2010; E. A. Casey et al. 2016; Flood 2011; K. C. Macomber 2012; Peacock and Barker 2014).

In this article, we present findings from an ethnographic pilot study with male allies involved in a violence prevention program in the informal settlements of Dharavi in Mumbai, India. The intervention was designed and implemented by the Society for Nutrition, Education, and Health Action (SNEHA), a NGO based in Dharavi and other informal communities in Mumbai. We look at the ways in which these young men’s participation in the intervention engenders transformations in their conceptions of masculinity and their personal and communitarian relationships. We also explore certain limits to men’s engagement that reproduce gender inequality or fail to critique men’s privileges. We argue for a contextual and relational approach to engaging men, which foregrounds critique of patriarchal structures and an engagement with women’s sustained anti-violence work to build accountability. Our findings are also the basis for designing a context-specific module for engaging men in an ongoing community mobilization VAWG prevention program.

## Engaging Men: Interventions, Allyship, and Challenges

A global review of programs that engage men and boys, published by the WHO in 2007, shows evidence of change in men and boys with regard to sexual and reproductive health, their use of violence against women, and their health-seeking behavior as a result of relatively short-term programs (Barker, Ricardo, and Nascimento 2007). Of the fifty-eight programs reviewed, seventy-seven were considered to be “gender-transformative.” That is, they aimed to change the “relations, norms, and systems that sustain gender inequality and violence” (Barker, Ricardo, and Nascimento 2007: 4; Jewkes, Flood, and Lang 2015). In the Indian context, programs with men have engaged in peer education and awareness (Verma et al. 2006), involved local coalitions of activists and activist-inspired groups (Das et al. 2012), and mass-media and awareness campaigns (Lapsansky and Chatterjee 2013), and have shown success in promoting gender equitable norms among men. Global exploratory research with men engaged in anti-violence activism has shown that male allies emphasize the importance of personal relationships with women as well as with other men and their engagement in other social justice movements as important pathways to activism (E. Casey and Smith 2010; E. A. Casey et al. 2016).

Most studies, however, do not provide deeper understandings of the social contexts in which men become engaged and the sorts of shifts, negotiations, and changes that such work engenders in them, in their conceptions of masculine identity and subjecthood, and in their communities. The level and degree of transformation achievable in such programs is also limited since they are “transforming or changing the social norms of a relatively limited group of men and boys and their partners and children” (Barker, Ricardo, and Nascimento 2007, 11). A focus on changing attitudes through pedagogic instruction is rather limiting because it “neglects the structural and institutional inequalities that are fundamental in shaping men’s violence against women” (Flood 2015, 164). Even among male allies and activists involved in gender justice and anti-violence movements, engagements tend to reproduce gendered hierarchies in how women’s labor is marginalized and how masculinity is redefined, instead of critiquing or challenging them (K. C. Macomber 2012). There is also limited insight to be gained into the active processes that male allies in the global South engage in, especially in engendering accountability to women in gender justice movements (Gilbertson 2018).

Contemporary sociological and anthropological scholarship in India has attempted to address the question of men’s engagement in feminist movements more concertedly, in terms of male scholars’ and activists’ participation in feminist mobilization and politics (Chowdhury and Al Baset 2015), and the reframing of masculinities and men’s subjecthood (Chopra 2007; Gilbertson 2018). Chopra’s (2007) edited work on men’s supportive practices and gender justice activism shows how allies are able to negotiate the complexities of masculinity, patriarchy, and violence and engender supportive relations and positions in families and kinship networks (Chopra 2003). Gilbertson’s (2018) recent ethnographic work with young men involved in gender justice activism in Delhi highlights the different levels and strategies through which young men partake in activism, such as education, awareness, and street theater. She notes, however, that for many male activists, the problem of patriarchy and violence against women is seen as one of “mind-sets” rather than of men or masculinity, framing issues of violence and inequality in depoliticized and individuating terms.

## Research Context

SNEHA is an NGO that emerged from the voluntary efforts of doctors associated with the Lokmanya Tilak Municipal General (LTMG) hospital, located in Dharavi, in 1999. LTMG outreach and maternal health-care centers were the focal point for SNEHA activities, which initially aimed to improve maternal and newborn health. However, frequent cases of domestic violence highlighted the need for sustained effort to provide help to vulnerable women beyond emergency medical relief. This was the beginning of SNEHA’s Prevention of Violence against Women and Children (PVWC) program, which began by establishing a crisis counseling center at the LTMG hospital Urban Health Center as well as recruiting women from Dharavi as voluntary frontline workers over the years, known as “sanginis” (Hindi, noun; feminine for “companion”). At present, the PVWC program has expanded in various localities in Dharavi and other informal settlements across the Mumbai region (Daruwalla et al. 2009, 2017). Informal settlements account for nearly 41 percent of all households in Mumbai (Chandramouli 2011) and are characterized by high population density and poverty. Basic infrastructure and services such as water, sanitation, and electricity are often lacking or provided illegally. While informal communities such as Dharavi are also marked by sociocultural diversity and are informal economy hubs (Sharma 2000), women and girls often face marginalization, domestic abuse, violence, and neglect (Datta 2012). The PVWC program’s work with sanginis and women’s groups has played an important role in transforming social relationships in the community and giving women collective agency and autonomy (Chakraborty et al. 2017; Daruwalla et al. 2015).

Along with sanginis and women’s groups, SNEHA also works with men from the community. Over the years, the program has used various strategies to engage men, such as the Dharavi Federation, an autonomous citizen’s group with male and female members that works on civic issues and infrastructure. Conversations around gender and VAWG were introduced gradually, linking them with women’s situations in their areas. Recently, the program has started engaging men in its violence prevention program by forming men’s groups on a pilot basis. At present, there are five groups in Dharavi. The pilot used the *Yaari Dosti* (Friendship) training manual on gender, health, and violence developed by the Population Council, in collaboration with Instituto Promundo, Committee of Resources Organisations for Literacy (CORO), and other community-based organizations in India, to engage young men and boys in HIV prevention and promoting gender equity (Verma et al. 2006). The manual is an adaptation of *Program H: Working with Young Men*, originally developed by Instituto Promundo, and includes discussion-based sessions covering gender, sexuality and reproductive health, violence, and living with HIV/AIDS and its prevention. The purpose of using the manual was to assess whether a direct gender transformative approach could lead to changes in the attitudes of men rather than involving them in civic issues to get them to start talking about gender and violence.

## Method

Between January and June 2017, we carried out a comparative study of women’s and men’s groups in Dharavi. This was framed by our previous research work in Dharavi, including a pilot study on sangini frontline workers (Chakraborty et al. 2017), in which we were concerned with the sustainability of the “sangini model” and the factors that motivated them. Consistent with some of our past research, we used ethnographic methods in addition to other qualitative methods such as focus group discussions (FGDs) for data collection. One of us (Chakraborty) spent four to five days a week with the community team members conducting participant observation and attended meetings of women’s and men’s groups. These meetings were interactive and would typically last between forty-five and sixty minutes. We observed forty-five such meetings (thirty with women’s and fifteen with men’s groups). Meetings with men were conducted at an empty office space (on Sundays), at a community center (in the evenings), or at an empty police beat office. The sessions usually involved the PVWC staff discussing a theme or topic from a series of modules from the *Yaari Dosti* manual.

After two months of preliminary work, we purposively sampled six women’s groups and three men’s groups with whom we conducted FGDs. In this article, we use data gathered from FGDs with a total of twenty male participants aged eighteen to thirty-five years. Those in their early twenties were students, and the others worked in semiformal jobs in the service or technical sector. Most of them had studied to higher secondary level (grade 12) and had been attending the men’s group for ten to twelve months. Most of them were also involved with local communitarian and political youth groups, where they volunteered and helped organize religious and social festivals. They were viewed as currently nonviolent (although some participants disclosed past abusive behavior with partners and some were seeking counseling services along with their partners). We obtained written consent from all participants before conducting the interviews. Quotations are pseudonymized.^1^

Transcripts were read several times and coded manually to generate themes, which were also informed by our observations from ethnographic fieldwork. The initial set of themes included participants’ reflections on being part of groups and networks; their notions and experiences of sexuality, sex, and gender; their engagement with ideas of masculinity, their understanding of VAWG; and their experience of change. On further review, we synthesized these themes into our three main findings: the production of individuated “good” subjects, tensions and ambivalence in their negotiations of masculinity, and the intersection of gender, space, and VAWG.

## Findings and Analysis

### Men’s Responses to Interventions

#### “We learn to become good men”: Impact of knowledge and awareness on subjecthood

When we began talking with our participants about their engagement and learnings in the program, their initial responses tended to highlight the importance of “knowledge” and “awareness,” which they felt led to discernible changes in their attitudes and perceptions. In most cases, awareness was linked with ideas of sex and sexuality, which were the topics that were covered in detail during the research period. For many men, being involved in the intervention was seen primarily as a way of acquiring knowledge about topics with which they had not normally engaged or had engaged with in different, nonpedagogic ways such as informal conversations, on the Internet or social media. This was largely a function of the information- and awareness-focused themes in the *Yaari Dosti* training manual, which covered a range of issues from sex and sexuality to GBV, HIV prevention, labeling and stigma, and emotional well-being. It was interesting to note the perceived utility and credibility of such knowledge. For these young men, knowledge acquisition and “awareness” were linked to a process of becoming “good” people and having a good life, of which a tangible outcome would be the ability to “find solutions” to problems. One participant, Lalit, said to another newer group member in their FGD:

In our lives, what our goals are, what we want to do in the future, they teach us about all that. These are our beliefs, and we want to live according to those. What are the risks we face, and if that happens, then what do we do ahead. They tell us the solutions for that as well.

The “topics” they learnt about, Lalit explained, “increase awareness about how we interact with others; how we respect seniors; how a husband should treat his wife; how boys should view girls.” Because of the intervention, “knowledge” on sex and sexuality was understood outside gender-unequal or misogynistic registers of meaning within which young men often discuss them. Implicit in their knowledge was the notion that it was somehow more correct than their prior understandings, which they defined as “normal.” They also noted tensions and anxieties around sexual performativity that such “proper” knowledge was able to assuage.

Lalit: First, we used to think it [discussing sex] is dirty; we were ashamed of it. We couldn’t express it to anyone. We had no knowledge about that. But now we have the knowledge, so we can discuss it freely, without any inhibitions…. Earlier, we didn’t know. So how could we explain it to others?

Sachin: In a manner of speaking, we didn’t know anything; we would understand it normally. But now we know it is an important concept.

Lalit: At first, all we would think of was trying to woo a girlfriend; kissing her, pressing her breasts, making out, all of that. But yes, we were afraid of intercourse. What would happen?

Sachin: We were afraid. What would happen if she became pregnant? We could get jailed! But now sir [the facilitator] has explained it to us.

Lalit: How to have sex, how to use protection, all that we got to know coming here. Now when we do it, we will remember and know what to do.

Rakesh, a participant from another group, also mentioned how “practical” know-how about their sexual lives was an important aspect of the intervention. For instance, he had only heard of certain contraceptive methods before, without actually knowing what they were, but he got to learn about them in a more practical way, and that contraception “isn’t there for only ladies,” and men can also use protection such as condoms and “be aware of other methods to prevent pregnancies.”

In their group discussion, Lalit also summarized an important learning about “consent,” a topic most men did not normally reflect on.

If a girl says no, then we shouldn’t think bad things about her…. If I am trying to woo a girl, and she rejects me, then she must have some reason for saying no, or she doesn’t like me; there must be some problem. We got to learn that here, about how to respect a person’s feelings.

Acquiring knowledge was not, however, a one-way process. The young men were not passive learners but sought to use the “resources” beyond their participation in sessions. Some participants, like Sachin, Lalit, and Rakesh, said that they would often try to reach out to the facilitators personally by phone or outside the sessions with queries about their sexual health. They felt that this was an important factor in keeping them engaged in the intervention and motivating them to mobilize other participants for the sessions.^2^ For younger men, especially, the groups presented an “opportunity” to participate in their communities outside of, or in addition to, existing social structures. Sachin and Lalit’s group, for instance, was able to organize a plenary on gender equality and women’s empowerment during a festival, to which they invited local women leaders as key speakers including a municipal corporation representative. Another participant in a different locality, Karan, who was also a member of a political party’s youth wing, emphasized the important function of “group bonding” that men’s groups are able to achieve, since being a part of groups means that “the message that’s going to [only] five to ten people, or to five people…should [also] go out to as many people outside as possible.”

We realize that such responses are typical in community-based interventions. They are similar to what we observed with women’s groups, from which women reported an increase in knowledge, awareness, and courage to discuss VAWG and intervene in cases of abuse as the most evident forms of personal and collective transformation (Chakraborty et al. 2017). However, although most participants reported an increase in awareness and knowledge on issues such as gender equality, it still tended to be discursive and generic and lacked any specific or experiential understanding, as women’s engagements often do.

#### Us and them: Individuating effects of the intervention

The intervention was able to give these young men the space and opportunity to become “good” subjects, but it did so in a manner in which the acquisition of “knowledge and awareness” had an individuating effect. This was at least partly a function of the morally and factually “correct” knowledge that came from a legitimate source such as an NGO. These individuating effects are analogous to the process of “subjectivation” as discussed in post-structuralist social theory (Butler 1993; Foucault 1990). Subjectivation refers to the idea that individual actors constitute themselves as ethical and moral subjects on the basis of relations of power and knowledge. Such power relations, Foucault (1990, 29) suggests, are not repressive but productive, in that subjects’ exercise of agency often involves the production of their very identities as objects, legitimized by laws or sets of laws to which subjects submit themselves.

In the specific context of VAWG interventions, this effect is individuating precisely because the form and content of knowledge under discussion—sex, sexuality, emotions, and violence—are reposited in individual registers of embodiment and agency but preclude engaging with collective responsibility and accountability. A corollary is the understanding that men who do use violence against women are *not* “good” subjects because their violence is a function of their lack of awareness: they are incomplete subjects. Accordingly, one of the causes of domestic violence was seen as a “lack of awareness” among both partners. One participant said, “both need to be at the same level. So when both of them are taught that [abuse is wrong], then abuse will reduce.” As Lalit said, “both [men and women] should have the understanding. *Men should also be aware of this*.” In other words, individuation is linked, in a collective sense, to perception of membership of an in-group (men who know) and the constitution of an out-group (men who do not know).

Similarly, violence and patriarchy are understood by their individuating actions and manifestations rather than as structural issues, as something that men who are not aware or knowledgeable do and not a product of larger social relations between men and women that are historically and politically shaped. This echoes K. C. Macomber’s (2012) insights into how male allies tend to reconstruct a particular form of masculinity in their engagements, as actualizing their “true self” as men. She writes,

Collectively, male activists taught each other about the damaging effects of traditional masculinity, and together they redefined what it meant to be men. From their point of view, they were living more authentic lives, not only as men, but as better men. (p. 35)

It also follows the conclusions that Gilbertson (2018) draws from her ethnographic research with young male activists who tended to frame the problem of patriarchy as an issue of “mind-sets” rather than structural or historical systems.

At the same time, it was not that the participants were unaware of the structural elements of violence and patriarchy: they would often overdetermine the individuating aspects of the structure. This was exemplified in conversations in which gender norms and patriarchal social mores were seen as emanating “from us” or that “these have been made by us”—a refrain that many of them would resort to when a facilitator asked them why gender unequal norms exist. As Rakesh explained, “Gender…[is] what society (*samaaj*) has made…. But who does the most work? The women do. Who has decided this? We have, together [decided] that women should do this work.” These relatively straightforward statements have been useful in the early stages of the intervention to explain the social construction of gender and make participants critical of reductionist assumptions about women’s inherent inferiority or the normalization of VAWG. When contextualized in the individuating effects of knowledge, however, they tended to prevent critical reflection on the structural nature of gender inequality in everyday life and men’s complicity in it. Indeed, when discussions veered toward examples from everyday life, some participants would fall back on gender unequal and misogynistic idioms and language, which are rooted in specific hegemonic constructions of masculinity for young men. We consider these next.

### Negotiating Masculinities

#### “My masculinity is”: Constructing “good” and “bad” masculinities

At a group session one Sunday afternoon, we were talking about ‘masculinity’ and violence with Sachin, Lalit, and other members in their group. They were clear that violence is wrong, but, as men, they insisted that if someone besmirched their “reputation” [in English] the use of physical violence was justified, so that “others know not to mess with us!” Respecting another’s reputation was central to their conception of masculinity. At one point in the discussion, they framed “their” masculinity as “good” or “real” [as opposed to “bad” or “false”]. When asked to reflect more, they suggested that the idea of maintaining one’s reputation—and, thus, respecting another man’s reputation—was central to “good/real” masculinity; “false” masculinity meant not respecting said reputation. Disrespecting women and other forms of gender unequal behavior like street sexual harassment were also seen as an aspect of “bad/false” masculinity, but appeared to be predicated on the idea of protecting women. If women reacted to men in a hostile manner, it was thought to be unfair, especially against those who adhere to “good” masculinity. (Field notes, February 2017)^3^

This vignette foregrounds one of our key thematic findings with regard to the overarching theme of masculinity, that of *ambivalence*, which can be understood in terms of further individuating effects of “awareness” and “knowledge.” During the above conversation, we felt that these men did not readily dismiss violence: it was still a legitimate issue. But it appeared that, despite violence being so prevalent, there was still a sense that it was important for women to perform docility—and not respond in anger—for their claims to be taken seriously. Our fieldwork with the rest of the men’s groups underscores some of the other factors that produce such ambivalence.

To return to the framing of “good/real” and “bad/false” masculinity, it is important to recognize it as being constructed by young men in a way that is still critical of biologically essentialist constructs of masculinity (for instance, attitudes regarding sexual virility and performance). Their critical reflections, however, often fell short. In another meeting of Sachin and Lalit’s group, we observed a brief conversation about a local woman who was described as promiscuous. At one point, Sachin remarked, laughing, that “she has one too many *kela*!” (banana, or in this case, penis). We pointed out that this was an instance of “labeling” someone negatively, which they had all said was problematic just a few moments before. Sachin and the others agreed but insisted that this woman herself “verbally abuses others.” When we tried to broach this topic in the FGD, they offered a more measured response:

Sachin: People say [things] like that a lot, that this woman eats too many bananas (*kela*)…

Lalit: So we can try to make the person understand. [We can ask] “Did you see it yourself? If not, stop. She can do whatever she wants. If you or I couldn’t woo her, then should it be that she has to have a boyfriend? She may have some different issues, and if she doesn’t want to discuss that, we cannot force her to…whether she wants to talk to us or not, she can decide that for herself.”

Jayant, a participant who attended sessions at our community center, was less ambivalent in his assessment of masculinity. Among our interviewees, he was someone whose contributions to the focus groups could clearly be considered as “benevolent sexism” (Glick and Fiske 1997). When we asked questions about masculinity and emotions, Jayant said, “but if a man has a daughter, mother, or sister-in-law, won’t he have to cry [if something were to happen to them]?” On gender equality, he said, “women are okay in [their] place…If they stay within their limit it is for their own benefit.” Limit, in this case, included “doing housework, making tiffin for [her] husband…bringing kids back from school.” It was “not wrong” for women to work, he said, but “doing something apart from a job is wrong,” probably referring to extramarital affairs that women could have—a topic of contention in many such meetings, presumably since this violates the assumption of legitimate domesticity (Datta 2012) that women are expected to inhabit even as they move out for work and employment (Roy 2003).

Harish, a former youth volunteer who also attended this group session, was the only one to challenge Jayant on this; the other participants kept more or less quiet. In fact, Harish was one of the very few participants whose allyship appeared to transcend the aforementioned individuating effects. When Jayant spoke about having “sympathy” for his mother and sister because they did all the housework—which is why he said he helped them “lift heavy weights”—Harish called him out.

Listening to [Jayant], don’t you see how he still believes in gender [roles]? “How will she lift such heavy weights?” So we see gender in this mentality…. If he thinks, “How will she lift?” then that is also gender, isn’t it? “She’s a woman, how can she lift such weights? I am a man, I can lift the weight.” Instead, it should be, it’s my responsibility. She always does this work; I should do it too.

When we brought up the question of whether “good” or “bad” masculinity exists, Harish said that “according to society, masculinity is that which keeps women under control” and that it was the responsibility of male allies and youth volunteers to reshape masculinity into one which seeks to engender equality rather than violence. He continued, “so that’s an idea that is created by society, that a man should never cry, and that women are restricted to only the stove and domestic boundaries, and keep suffering.” Much like the narrative of change that other participants noted, Harish said that the NGO facilitators had played an important role in this and that he realized that it was important for there to be “trust-bonding” between him and his peers and added “it is my responsibility that I don’t let that bond break.”

#### “How do I go ahead?” Ambivalence, hegemony, and tensions

This form of “trust-bonding,” however, is much harder to achieve for others like Rakesh and his friends, since many of them are “frustrated” in life—about their socioeconomic conditions, debts, familial responsibilities, and concerns about their future. Rakesh said that young men “wished that there is balance, in daily [life]…that they live peacefully.” Hemant, another group member, said,

We ourselves are not happy in our lives, and we are not settled…. We have no satisfaction ourselves, so how do we handle other people’s affairs? (…) So it’s happening like that, we are trying to work for the uplift of society, but when will our upliftment happen? When my home is all right, only then will I go to uplift others. When I am stuck in my problem, so how will I take a step ahead?

For young men like them, they reasoned, reflecting about masculinity and working for gender equality is relatively difficult to achieve in everyday life because of material constraints and anxieties. At a deeper level, it was also difficult for them to think of change or awareness because of their “frustrated” condition, which Rakesh said was akin to being “trapped in violence.” He added, “So how do I help others in violence?”

How do such forms of awareness coexist with problematic and sexist attitudes that do not fully recognize the inequalities of gender, or recognize them only partially, to a point where men’s privileges are not questioned? How do such experiences of change and forms of allyship coexist with material insecurities, anxieties, and frustrations? How does a “good–bad” binary notion of masculinity function in a context in which some men cannot formulate a discursive or material engagement with masculinity itself?

As noted in the masculinity studies literature, heterosexual masculinities are framed in culturally specific forms of “hegemonic masculinities” (Connell 2005), which account for the intersectional relations of power between different and *multiple* forms of masculinities, based on the hierarchical gender relationships between women and men. Building on the work of Gramsci, “hegemonic masculinity” refers to the “masculinity that occupies the hegemonic position in a given pattern of gender relations, a position always contestable” (Connell 2005, 76). Apart from hegemony, the other forms of masculinity outlined by Connell include “complicity,” “subordination,” and “marginalization” (pp. 78–81). Any form of masculinity is hegemonic insofar as it embodies a “currently accepted” strategy—it is open to challenges and changes; it is a “historically mobile relation” (p. 77). While hegemonic masculinities do not necessarily exist in the actual lives of men, they “express widespread ideals, fantasies, and desires…they articulate loosely with the practical constitution of masculinities as ways of living in every-day local circumstances” (Connell and Messerschmidt 2005, 838).

With men involved in gender justice movements or interventions such as ours, however, an *ambivalence*—recognizing that VAWG is “wrong” but reconstructing “good” forms of masculinity that come with expectations of legitimate domesticity and gender politics—is crucial to theorize the transformation or negotiation of hegemonic masculinities within VAWG prevention and how masculine powers and privileges are reproduced in different ways. This also follows scholarship on ambivalent sexism as proposed by Glick and Fiske (1997), which suggests that alongside more “hostile” forms of sexism, there also exists “benevolent sexism,” which “relies on kinder and gentler justifications of male dominance and prescribed gender roles; it recognizes men’s dependence on women (i.e., women’s dyadic power) and embraces a romanticized view of sexual relationships with women” (p. 121).

Following the work on ambivalent sexism, and Connell’s influential scholarship on hegemonic masculinities, we propose to take the ambivalence in our participants’ discourses and narratives more seriously. We argue that ambivalence is an important way of making sense of how these young men negotiate multiple masculinities in their everyday life and how ambivalent sexism is often folded into such negotiations. Masculinity is ambivalent because it includes combinations of hegemonic, complicit, subordinate, or marginalized masculinities rather than just one or the other. In fact, it shows the permeability and fluidity of these categories, which are “not…fixed character types but configurations of practice generated in particular situations in a changing structure of relationships” (Connell 2005, 81).

Jayant’s explicit benevolent sexism coexists with Rakesh and Hemant’s “frustrations,” with Sachin and Lalit’s failure to critique sexism and Harish’s well-developed sense of allyship. The kinds of masculinities these men discuss and attempt to construct are themselves specific outcomes of the intervention—which could be said to construct a set of hegemonic masculinities, like that of an “ally” or “responsible” men, but who are still viewed as bearers of social power and respect. It is here that tensions between sexism and equality, anxiety and accountability, trust and frustrations become salient. It is ambivalent precisely because it is unfinished, fragmentary, in the process of becoming. As our evidence suggests, in order to critically evaluate men’s participation in anti-violence work, we need to look at the social and cultural formations of masculinities that are situated in everyday life, within which ambivalent sexist attitudes are contextualized, sometimes challenged, even as men continue to negotiate their constraints.

### Gender, Space, and Violence

#### “A space to be oneself”: Creating private spaces to engage men

In this section, we specifically examine how spatial and material conditions in informal settlements intersect with gender relations and VAWG. We refer to actual sites in which our intervention takes place, as well as the wider spatial and sociopolitical context of urban informal settlements in India. Particularly important in our analysis is the division that social actors draw between private or intimate spaces and public spaces with regard to gender, family, and politics of urban commons. The discussion in this section is based on our ethnographic evidence and interview data, but we also draw on our previous work with our women frontline workers (sanginis) to contextualize men’s experiences and negotiations of this public–private divide. Like our emphasis on subjecthood, individuation, and masculinity, we argue that the attention to spaces—private and public, intimate and common—demonstrates how VAWG prevention interventions produce certain effects among recipients that are vital in contextualizing the changes and transformations they are able to engender in their social landscapes.

The availability of some form of private or quasiprivate space was crucial to sustaining the program, as a gathering place for men where facilitators could talk with them and they could talk openly. This differs from the kinds of urban space that Chopra (2003) discusses: places where men hang out which have an intrinsic social value for social reproduction through friendships and supportive practices, a fact neglected in earlier scholarship on masculinity in South Asia (pp. 1654–55).

Despite men occupying such public spaces, there is still a need in our context to create private space to allow them to discuss issues such as sex and sexuality in a manner different from their prior understandings, stepping away from the abusive or sexist language of their “usual” spaces. The intervention opened up a space for men to “hang out” *outside* their everyday spaces, where they could talk about issues which would often deviate from “traditional” masculinity scripts or negotiate a space for themselves outside the hierarchies of age. Unavailability of space would often mean that sessions would be postponed or canceled, as participants were often unwilling to talk in public spaces such as street corners or the thresholds of their houses.

The session was held in an *aanganwadi* (preschool) since the police beat where we often met was unavailable. We had formed a circle and were seated on the floor. The facilitator asked the group to review what they had covered so far, and if they were observing any change in themselves. One participant, Naresh, in his late 30s, spoke in broken Hindi about how his relationship with his wife and children had transformed once he started attending the sessions. He had not been interested in the sessions initially (his friend had invited him), dismissing them as a waste of time. As the weeks passed, and with his friends talking to him, he said that his attitude changed as he began to be more open in communicating with his wife instead of neglecting her, and started sharing chores and looking after the children. This was the first time he had ever shared what he felt; he never had the space or opportunity to do so. The mood was silent, but contemplative; we observed appreciative nods all around. (Fieldnotes, February 2017)

We met in a participant’s house because there was no space available that Sunday. This time the topic was violence against women. Unlike the last session, the mood was quieter; the air hung heavy with silence and heat. Their responses were short and they spoke softly. Women face a lot of pressure, they said, from their homes, from society, from the community, and the family. “But what can we do?” was their question. The facilitator suggested going with their group to help out someone in distress. “What do we do if the perpetrator keeps abusing?” Rakesh asked. “What if they don’t want to call the police…?” The facilitator said, at that moment, if we are able to save even one woman’s life, we should not hesitate to do so. Toward the end, the mood was still solemn; there were no appreciative nods, as most participants had their gazes fixed on the floor. As we left, one participant, Pratik, remarked with a sense of appreciation, “It feels like you will make us cry, sir.” (Field notes, April 2017)

Such attention to the creation of nondomestic private spaces as sites of intervention, where men can talk about gender inequality, VAWG, and the experience of change, brings to attention how men try and make sense of gender inequalities and violence; how it opens up spaces for them to engage in affect and emotions, to negotiate vulnerability, especially in social contexts where there are few avenues for them to do so in meaningful and reflexive ways—to be made to feel like they would cry, for instance. The need for private spaces is not about catering to men’s needs or discretion (although this is part of it) but to understand and remap the public–private boundaries and their relation to gender hierarchies and arrangements in informal communities.

#### Negotiating public–private boundaries

While engaging men in anti-violence work requires a movement into the private, there are limits to achieving its requisite opposite: their engagement with the public with regard to preventing violence. An important reason for this is the individuating effects that interventions produce. Talk about change and violent behavior is still largely understood to be individual problems for a few men, even as some begin to reflexively acknowledge the structural nature of the issues. Another reason is that arrangements of public and private spaces structure gender relations and vice versa. Intimate spaces, which are coded as domestic and feminine (Gal 2002, 82), are important sites within which masculinities and femininities are renegotiated. There are limits to these, since the public sphere—which is often coded as masculine—is a site where men are *unable* to effect change in terms of gender inequalities or effect change in a way that redraws the divide between public and private, masculine and feminine. This is further complicated in cases of VAWG, where women are seen to require “protection” in public spaces, whereas violence perpetrated on them in intimate spaces is seen as “ordinary” (Datta 2012, 151).

For instance, before we began to engage men using the *Yaari Dosti* training manual, we would organize meetings with them where they could network with the SNEHA’s sangini volunteers and other local activists to engage in civic issues. In one meeting, we observed in July 2015, the discussion on civic issues was explicitly and quickly politicized. Male participants brought their public personas and political affiliations into the private space created for reflection and deliberation, somewhat neglecting notions of support and accountability. In a sense, this illustrates the historical creation of normative orders wherein masculinity, public spheres, and politics tend to align and exclude women (Roy 2003, 86–87; also Gal 2002, 81–82). In informal settlements, furthermore, the boundaries between public and private are already rendered fluid because of their built forms. Households, thresholds, alleys, and streets form a spatial continuum rather than discrete entities. In such cases, gender relations become vital to maintaining boundaries between the home and the outside and within the domestic space. Women’s lives, experiences, and narratives are rendered invisible in such discourse, subsumed into the domestic realm (Roy 2003).

Our work with women’s groups, however, destabilizes this public–private boundary by framing VAWG and domestic violence as issues that cross it. Unlike with men, our work with women is folded into their everyday lives, where women politicize “the home through discussions and dialogues on feminist issues” to raise “feminist consciousness” (Datta 2012, 93–94) and also intervene in public. In our previous work, this was exemplified in a case in which a group of sanginis were instrumental in mobilizing against an illegal cellphone tower in their neighborhood (Chakraborty et al. 2017, 1348). They framed the tower as not just a hazard to physical health but as a risk that would disproportionately affect other urban poor women, children, and elders. Similarly, in issues of VAWG, women emphasized the need to “join families, not break them,” but recognized that their “job is to resolve the issue and not increase the violence” (p. 1349). Actions like these place women as important urban actors who draw on metaphors of care and emotional labor to prevent violence and intervene in urban issues, showing that “the contours of what are considered as private and public are continually problematized and remapped.”^4^

Contrary to this, most of our work with men has been successful enough to, mainly, create private spaces in which men can reflect and learn in a way that their presence in public spaces prevents them from doing—as we saw with Rakesh and Hemant’s group. The creation of such spaces is not neutral; inasmuch as opening up spaces for men to engage in emotional work is important, engendering change and accountability toward women is also crucial and nonnegotiable. This is precisely where we see important limits.

One way we tried exploring the idea of public engagement was through the issue of bystander interventions. In their FGD, Sachin and Lalit recounted an incident in which they and their friends intervened when a young woman was being physically abused by her partner. When they intervened and used physical violence against the perpetrator, the girl said, “it is between us! Why are you interfering?” Lalit reasoned that it was important to discern whether “violence is really happening.” If not, intervening would be “the same as harassing her, since it is being done by force.” Sachin added that in such cases, women may create another scene, and there is a fear that “nothing should come onto us.” Lalit continued, “Yes…if this is happening to a girl, then ten people will appear, and try to solve it. But if something is happening to a guy, then the public will come and hit that guy first!”

It is understandable that volunteers, whether men or women, would probably lack confidence to engage in bystander interventions at an early stage. The risks of harm to oneself or one’s family are real, and support systems such as police or social workers are often lacking. However, the above interaction specifically frames the difficulty of bystander intervention as a *gendered* problem. The responsibility, our participants felt, lies with the woman who consents to violence and is unappreciative of the person intervening, usually in the role of a male protector. As Sachin said, men may not have any issues in getting involved in altercations or even using physical violence against perpetrators. What stops them is that women survivors might not appreciate their protection and that such issues, even as they take place in public, are still considered “private matters” by survivors, and those interested in intervening do not sufficiently interrogate this.

Alongside knowledge and awareness, space is also central to the problem of individuation. It is where men’s subjecthood as allies is negotiated but constrains their ability to act, since acting would require moving out of quasi-private spaces and actively upending the structures that confer them privileges. In other words, spaces, for male allies, are either *too intimate* to affect outward change or *explicitly political*, which forecloses any engagement with the domestic, with the two never really aligning. This is perhaps the most significant aspect of space when we think of how it intersects with masculinity and violence prevention work and how it contrasts with women’s engagement with VAWG and other public issues.

## Discussion: Possibilities and Constraints on the Path of Allyship

Our experience of engaging men and working with male allies has been interesting but challenging. It is now an important and inevitable aspect of VAWG prevention programs, but there are limits to the existing literature, especially in its emphasis on changing men’s attitudes and behaviors. We have two ideas about ways in which our findings can help us frame a more sustained engagement with men that recognizes their subjective experiences but also seeks to engender accountability in anti-violence work.

While we concur with scholars like Chopra (2003, 2007) who argue that social research should take seriously men’s current supportive practices, we believe that men’s engagement in anti-violence work should further be contextualized within the care and emotional labor that women perform to prevent VAWG in their communities. As we saw, men’s experience of transformation and change is relatively instrumental and incremental compared with women’s. The laborious task of mobilizing a few men to attend group education sessions, compared with the relatively easier task of mobilizing groups of women, puts expectations and pressures on the program.

This raises the question of whether the challenges of working with a few men are worthwhile, given the likelihood of individuating effects rather than community transformation. Although we think that both are important in their way and can bring about change, depending on program vision and resource availability, the question remains: how do we find a balance between paying attention to men’s vulnerabilities and critiquing hegemonic patriarchal practices? We recognize that the mainstay of interventions should be consideration and criticism of gendered power imbalances to ensure that, in addition to supportive practices, notions of accountability are addressed through a holistic and relational approach involving women and men. This would ensure that interventions prioritize the needs of women and girls in communities and engender care and empathy among male allies as well, so that the undue burden of care and support do not fall on women in the intervention or in their families and communities.

As researchers and practitioners, we also feel that our insights extend beyond our participants to the local and regional context of gender activism in India, within which we have noticed that many senior male activists appear unaware of them. By emphasizing the singularity of men’s experiences, programs and public outreach have arguably tended to reproduce the individuating effects we have described. This means that conversations have often been about “some good men,” whereas women’s activists continually emphasize “women’s lives” to underscore their collective experiences of violence, change, and transformation. We need to consider critically the costs of and limits to allyship and engagement, especially in contexts where women’s grassroots organizations have to compete for funding to sustain their work with survivors of VAWG. The Coalition of Feminists for Social Change (COFEM) have said recently that efforts to engage men often lack a feminist analysis, draw attention away from the structural nature of VAWG (COFEM 2017b, 2017c), and lead to lack of accountability in program development and policy frameworks (COFEM 2017a). We propose that conversations among activists and social organizations must address the question of accountability in engaging men (K. Macomber 2015). Paying attention to men’s needs and anxieties must be accompanied by an understanding of the emotional labor involved in women’s efforts to prevent violence and address other gendered issues We are addressing these important issues in our ongoing work, in which we have incorporated insights from the research to ensure that individuating effects are minimized in men’s engagement in VAWG prevention. We have revised our training manuals and mobilization strategies in an effort to ensure that male allies are accountable to the women in their communities and participate actively in support and care practices.^5^

## Conclusion

We have examined our ongoing work with young men in urban informal settlements to understand important aspects of their engagement and negotiation with an NGO violence prevention program as well as their experiences and negotiations of masculinity, subjecthood, gender, and space. Our findings point to a need for closer and contextual examinations of men’s lived realities and material conditions rather than to focus on changes in their attitudes and behaviors. Young men negotiate multiple masculinities in their social contexts, which are at once hegemonic and ambivalent, and within which transformation is accompanied by persistent sexism and socioeconomic frustration. In examining the spatial configurations of informal communities, we show that a limitation to men’s involvement in anti-violence work is their inability to transcend and critique the public–private divide and understand VAWG as a public issue. Since engaging men has become an important component of interventions across the world, we argue that it must acknowledge the historical labor implicit in women’s efforts to prevent violence and that it bears the responsibility for accountability.

## Notes

1. The study was included in formative research for intervention development, approved by the Ethico’s Independent Ethics Committee, Mumbai on December 2015.

2. While National Family Health Survey-4 data for urban Maharashtra show that nearly 90 percent of men are aware of the fact that condom usage prevents HIV transmission (Indian Institute of Population Sciences & Ministry of Health and Family Welfare 2017), several participants in Lalit and Sachin’s group reported a degree of uncertainty about condom usage when they visited sex workers. Sometimes, they would engage in anal or oral sex with transgender sex workers. Prolonged conversations with the nongovernmental organization’s facilitators, however, tended to allow them to make more informed choices, since they became more aware of the risks of sexually transmitted infections.

3. This is an edited extract from Chakraborty’s ethnographic field notes and memos recorded during fieldwork. All vignettes are based on field notes.

4. We thank one of the anonymous reviewers for their comment which led us to strengthen this comparison.

5. These developments are part of an ongoing cluster randomized control trial of community interventions to prevent violence against women and girls (VAWG) in urban informal communities (http://www.isrctn.com/ISRCTN84502355). We have compiled a new training manual after reviewing and adapting global manuals such as *Rethinking Domestic Violence* (Raising Voices 2004), *Engaging Men and Boys in Gender Equality* (Acquire Project and Promundo 2008), and *Engaging Men in Accountable Practices* (International Rescue Foundation 2014), which focus on men’s emotional and mental well-being, healthy relationships and accountability, alongside issues of VAWG. We would like to thank one of the anonymous reviewers for their comment asking us to explain the relationship between this research and our ongoing work.
